# The Foegen effect: A mechanism by which facemasks contribute to the COVID-19 case fatality rate

**DOI:** 10.1097/MD.0000000000028924

**Published:** 2022-02-18

**Authors:** Zacharias Fögen

**Affiliations:** Theaterstr. 6, 34117 Kassel, Germany.

**Keywords:** case fatality rate, coronavirus disease 2019, facemasks, Foegen effect, Kansas, mask mandates, severe acute respiratory syndrome coronavirus 2

## Abstract

Extensive evidence in the literature supports the mandatory use of facemasks to reduce the infection rate of severe acute respiratory syndrome coronavirus 2, which causes the coronavirus disease (COVID-19). However, the effect of mask use on the disease course remains controversial. This study aimed to determine whether mandatory mask use influenced the case fatality rate in Kansas, USA between August 1st and October 15th 2020.

This study applied secondary data on case updates, mask mandates, and demographic status related to Kansas State, USA. A parallelization analysis based on county-level data was conducted on these data. Results were controlled by performing multiple sensitivity analyses and a negative control.

A parallelization analysis based on county-level data showed that in Kansas, counties with mask mandate had significantly higher case fatality rates than counties without mask mandate, with a risk ratio of 1.85 (95% confidence interval [95% CI]: 1.51–2.10) for COVID-19-related deaths. Even after adjusting for the number of “protected persons,” that is, the number of persons who were not infected in the mask-mandated group compared to the no-mask group, the risk ratio remained significantly high at 1.52 (95% CI: 1.24–1.72). By analyzing the excess mortality in Kansas, this study determines that over 95% of this effect can solely be attributed to COVID-19.

These findings suggest that mask use might pose a yet unknown threat to the user instead of protecting them, making mask mandates a debatable epidemiologic intervention.

The cause of this trend is explained herein using the “Foegen effect” theory; that is, deep re-inhalation of hypercondensed droplets or pure virions caught in facemasks as droplets can worsen prognosis and might be linked to long-term effects of COVID-19 infection. While the “Foegen effect” is proven in vivo in an animal model, further research is needed to fully understand it.

## 1 Introduction

The coronavirus disease 2019 (COVID-19) pandemic struck the world with over 228 million confirmed cases and over 4.69 million confirmed deaths worldwide by September 18th, 2021,^[1]^ resulting in a case fatality rate (CFR) of about 2.06%. The mortality rate of COVID-19 has been shown to increase with the overall mortality rate of the population.^[2]^ Mortality rate is the most commonly expressed measure of the frequency of occurrence of deaths in a defined population during a specified interval. However, the crude death rate calculates the number of deaths in a geographical area during a given year, per 100,000 mid-year total population of the given geographical area during the same year. Therefore, it is a better parameter to assess death rates among different populations.

Mandatory wearing of masks to cover the nose and mouth is a widely applied strategy in the management of the COVID-19 pandemic across many countries in the world. A lot of focus has been centered on the question whether mask mandates reduce infection rates. A study conducted in the Kansas state of USA showed a reduction in infection rates,^[3]^ while a Danish study did not find any protective effect of wearing masks.^[4]^

However, a lot less focus has been centered on the course of the disease while using masks. This is a questionable approach, as the question “how many lives can be saved?” is more important than the question “how many infections can be prevented?”.

Therefore, the aim of this study was to assess the influence of mask mandates on CFR by comparing the CFR between 2 groups, 1 with and the other without mask mandates. The corresponding two-sided hypothesis is that mask mandates change the CFR. While an increase in CFR may look unintuitive at first glance, more intuitively, one would not exchance his facemask with another person out of fear to breath in the virus that is caught in the facemask and get infected. Thus, breathing in one's own virus might increase the CFR.

The state of Kansas, USA has over 2.8 million residents. During the summer of 2020, Kansas State issued a mask mandate, but it allowed its 105 counties to either opt out or issue their own mask mandate – which was a rarity in the USA and 1 reason for the choice of this state, the other being that the comparison of infection rates among these counties has already been done by Van Dyke et al,^[3]^ showing a benefit of mask mandates.

Out of the 81 counties that had opted out and did not issue their own mask mandate, 8 large cities from 7 counties, had issued a mask mandate. This current study focused on the CFR, and whether mask mandates actually had an effect on the number of lives lost during the COVID-19 pandemic.

## 2 Method

This study applied secondary data on case updates, mask mandates, and demographic status related to the Kansas state, USA. As this is a secondary data analysis, ethical approval was not necessary.

A 3 + 3 step model was applied for the analysis of these data.

### 2.1 Step 1: Categorizing the counties into two groups

Using the information on counties with facemask-related regulations from the study by Van Dyke et al,^[3]^ which used data from the Kansas Health Institute and CDC, 105 counties were categorized into counties with mask mandate (MMC) and counties without mask mandate (noMMC). Further, the counties without mask mandate were evaluated to identify cities with mask mandates^[5]^ in them. Then the percentage of the county population^[6]^ that was represented by these cities^[7]^ was assessed in order to eliminate counties in which about half of the population was under a mask mandate, as they would dilute the results.

Thus, in order to guarantee that the cities with mask mandates constituted either more than twice of or more than half of the county's population not under a mask mandate, if more than 2/3 of these counties’ population was either under mask mandate or not, the county was included in the analysis and moved to the corresponding group. Correspondingly, if the city's population was within +/-17% of half of the county's population (that is, between 33% and 67%), the county was excluded.

### 2.2 Step 2: Parallelizing the groups

Since the assumption was close that counties with a more vulnerable population had issued a mask mandate (bias by selection), the specific COVID-19 risk of each group's population was assessed. The study by Vasishtha et. al^[8]^ demonstrates that COVID-19 mortality is closely matched with overall mortality, which is represented by the crude death rate (CDR) of any given population. The CDR represents age, pre-existing illness and all other mortality-bound cofactors in the underlying population.

Further, the CDR of each county for 2019^[9]^ was modified by subtracting deaths from causes that are clearly not a risk factor for COVID-19 to prevent statistical anomalies when comparing CDR, like an unusual spike in deaths from external causes or perinatal mortality in single counties. The following categories of the Kansas Health Institute death data were thus excluded to calculate a covid-related death rate (crDR): “pregnancy complications,” “birth defects,” “conditions of the perinatal period (early infancy),” “sudden infant death syndrome,” “motor vehicle accidents,” “all other accidents and adverse effects,” “suicide,” “homicide,” and “other external causes”.^[9]^

This crDR of the counties was then population-weighted (multiplied with population of county divided by population of group) and added up to calculate the crDR (total number of expected deaths per 100,000 people per year) of both the MMC and noMMC groups.

The assessement showed that, after step 1, the crDR of the noMMC group was 1012.6 deaths per 100,000, while the MMC group had an crDR of 782.5 deaths per 100,000, clearly indicating a bias of noMMC group being a more vulnerable population, counterintuitively.

**Figure 1:**
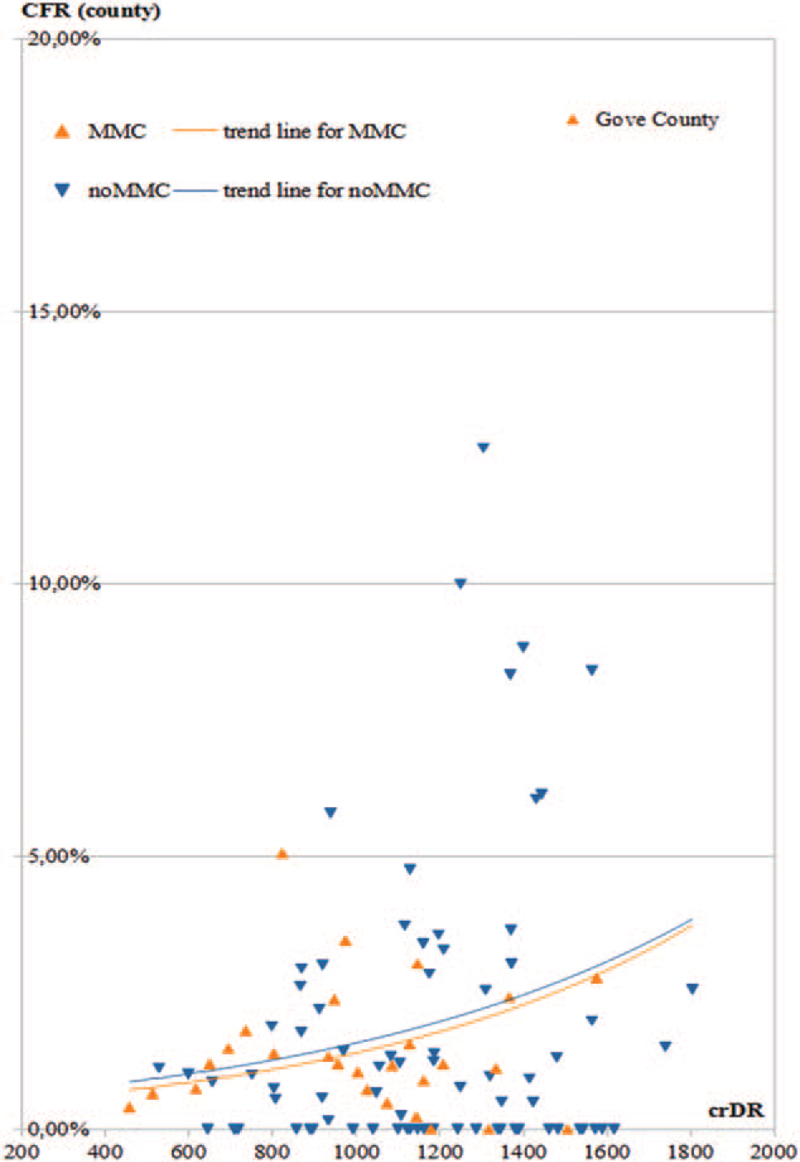
Scatterplot of COVID-19-related death rate (crDR) vs. case fatality rate (CFR). Orange triangles pointing upwards represent mask-mandated counties (MMC), blue triangles pointing downwards represent counties without mask mandate (noMMC).

Due to the lack of normality and homoscedasticity (as demonstrated in the scatterplot, Fig. 1), a regression was not possible, thus, the counties were parallelized for comparison based on crDR.

In this process counties were excluded until both groups had a matching crDR, meaning both populations are equally vulnerably to COVID-19.

This process of parallelization is a customized modification of the usual process used in parallel studies. It is based on larger groups (county populations) instead of individuals while likewise aiming to eliminate the aforementioned confounder.

There were 2 ways in order to get almost the same crDR in both groups:

A) Removing primarily counties with the highest crDR in the group with a higher crDR until both groups had the same crDR: Configuration A.
B) Removing primarily counties with the lowest crDR in the group with a lower crDR until both groups had the same crDR: Configuration B.

Therefore, cut-off limits of crDR were used in an attempt to reduce the crDR difference while trying to include the largest percentage of the eligible Kansas population.

### 2.3 Step 3: Analyzing the data

As the mask mandate was issued on July 3rd, August 1st was considered as the start date to allow for necessary adjustments to the mask mandate and prevent overlap with time before the mask mandate as the effect of mask mandates may not be visible immediately.

Moreover, October 15th was fixed as the end date as proof of mask mandates was available up to that point, and the existent mask mandates were revised after that date. The number of infected cases^[10]^ was calculated for this period.

The COVID-19 death count in Kansas^[11]^ is not personalized, meaning for each death counted there is no information on the person's infection date. After referring to the study by Khalili et al,^[12]^ the calculation of deaths was delayed to 14 days after the COVID-19 infection time period. In order to mitigate the influence of the start and end of the time interval, the number of deaths as the average of death differences between August 7th and October 22nd, August 14th and October 29th, as well as August 21st and November 5th was calculated. This way, both infection and death data were obtained for a span of 76 days. Based on these numbers, infection rates and CFR were calculated for both groups in both configurations.

A fourfold table was applied for the Chi-Squared test (α = 0.05) and risk ratio (RR; MMC to noMMC), and 95%CIs were calculated to determine whether the mask mandates significantly increased or decreased the CFR by COVID-19.

All statistical calculations were done using LibreOffice 7.1. (The Document Foundation, Berlin, Germany).

### 2.4 Step 4a: Infection rate correlated bias check (when applicable)

If the RR was significant, a sensitivity analysis is used to verify whether a difference in infection rate explains the difference in the CFR. For this, λ_low-CFR_ was considered the infection rate of the group with a lower CFR, and λ_high-CFR_ was considered the infection rate of group with a higher CFR.

The 2 possibilities were:

1. The group with low CFR also has a lower infection rate.
  If λ_low-CFR_ < λ_high-CFR_, there might be a testing bias.
  The hypothesis to this would be that if both groups had been tested equally and both had equal infection rates, the CFR would not be significant. In order to prove this hypothesis, the number of deaths in the group with a lower CFR was reduced by multiplying it with the factor (λ_low_ / λ_high_), the fourfold table from step 3 was revised, and a repeat calculation of the Chi-Squared, RR, and 95%CI was done.
2. The group with lower CFR has a higher infection rate.
  If λ_low-CFR_ > λ_high-CFR_, there might be a bias by protection.
  The hypothesis would be that if those protected by a reduced infection rate were counted as survivors (although they could still be infected later), the CFR would not be significant.
  In order to prove this hypothesis, the number of infected people in the group with a higher CFR was increased by multiplying it with the factor (λ_low_ / λ_high_), the fourfold table from step 3 was corrected, and calculation of Chi-Squared, RR, and 95%CI was revised.

### 2.5 Step 4b: Confounder check (when applicable)

If the RR was significant, further analysis was performed to find whether a confounder caused the RR (for MMC) to increase or decrease independently of severe acute respiratory syndrome coronavirus 2 (SARS-CoV-2) infection. This could be, for instance, the accumulation of fungal spores or bacteria in the mask or mask-induced hypoxia (increasing RR), or the prevention of other possibly lethal viral or bacterial infections (decreasing RR).

The hypothesis would be that a confounder in MMC causes increase or decrease in the RR independently from SARS-CoV-2. If this were true, the effect of masks would occur not only in the infected population but also among the not infected population under mask mandate. This can be proven wrong if the potential effect does not align with overall excess mortality in Kansas.

Therefore, it was necessary to calculate the additional deaths by mask mandates or the reduced death by mask mandates (for RR and both ends of its 95%CI as in step 3).

These additional/reduced deaths were calculated as the absolute value of

(1/ϕ – 1) ∗ death_MMC_

where ϕ is RR (or the values of both ends of its 95% CI), and death_MMC_ is the number of deaths in MMC. Further, the expected additional/reduced deaths (in all infected and non-infected) in all MMC counties were calculated by dividing by the number of infected persons in MMC (as obtained in step 3) and multiplying with the total population in all MMC (from step 1).

This result was compared to the (total) Kansas non-COVID-19 excess mortality during the corresponding weeks as already calculated by the CDC.^[13]^ The process involves calculating and adding up the difference between nonCOVID-19 deaths and the average expected number of deaths for each given week. The resultant value indicates the nonCOVID-19 excess deaths.

By dividing this number with the expected additional/reduced deaths in all non-infected in all MMC countries, it is possible to estimate the proportion of the RR increase/decrease calculated in step 3 that is not related to COVID-19 and thus indicating the influence of possible confounders.

### 2.6 Step 4c: Negative control (when applicable)

In case there is a difference after Step 3, the same group of counties would be analyzed using data from February 1st as starting date and April 15th as the end date for cases. The number of deaths was calculated as the average differences of February 8th to April 22nd, February 15th to April 29th and February 22nd to May 6th. These dates were chosen because shortly after April 15th, Kansas was hit by the 1st wave of the COVID-19 pandemic.

This resulted in multiple problems. First, case numbers increased rapidly and resulted in a strong undertesting, resulting in a test positivity rate^[14]^ of 18% on April 21st and 22nd, which then dropped consecutively due to massively expanded testing to 3.7% on June 7th, which is problematic as the positivity rate influences CFR. Furthermore, hospital capacity during the first wave was limited which may have resulted in medical undersupply and increased CFR. As the first wave hit all counties neither simultaneously nor in same intensity, I did exclude this timespan as it would incur massive bias.

As a comparison, during the chosen time span from Step 3, positivity rate was constantly between 6.9% and 9.9%.

## 3 Results

### 3.1 Step 1: Categorizing the counties into two groups

Figure 1 gives an overview of the mask mandates in Kansas counties.

Evaluation of the cities with mask mandates in noMMC is shown in Table 1.

**Table 1:** Large cities with mask mandates in counties without mask mandates.

City		County		
Name	Population	Name	Population	Population City/County
Emporia	24.765	Lyon	33.195	75%
Hays	20.852	Ellis	28.553	73%
Manhattan	53.678	Riley	74.232	72%
Marion	1.787	Marion	11.884	15%
Osawatomie	4.266	Miami	34.237	29%
Paola	5.670			
Parsons	9.665	Labette	19.618	49%
Winfield	12.057	Cowley	34.908	35%

**Figure 2:**
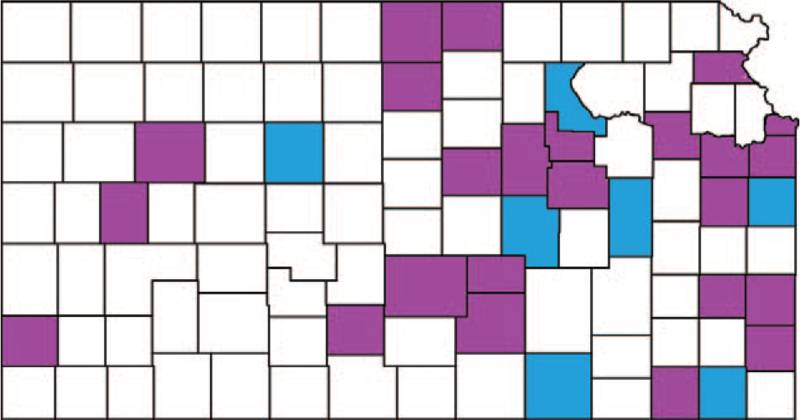
Mask mandates in Kansas counties. Counties with a mandatory mask mandate are purple, counties without a mandatory mask mandate are white. Blue counties are counties without mask mandate that have one or more larger cities with a mask mandate.

Figure 2 shows the result of these evaluations. There were 27 counties in the MMC group, 76 in the noMMC group, and 2 were excluded.

### 3.2 Step 2: Parallelizing the groups

**Figure 3:**
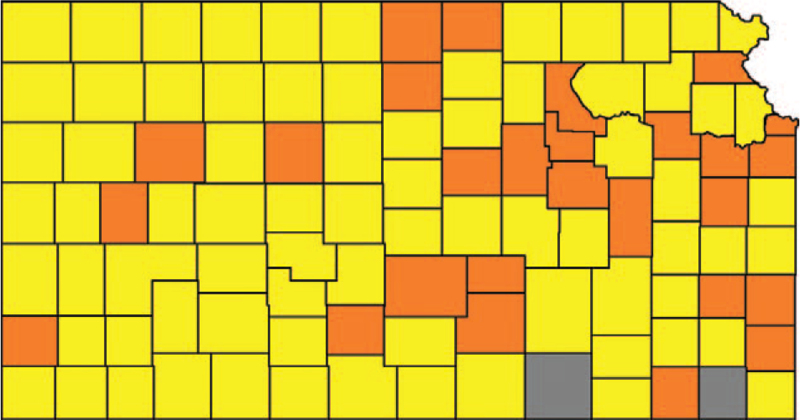
Counties after evaluating the major cities with mask mandates in counties without a mask mandate. Mask-mandated counties (MMC) are orange, counties without mask mandate (noMMC) are yellow. Grey counties were excluded.

Figure 3 shows the scatterplot of crDR and CFR by county and after step 1, the single outlier of Gove County (MMC) being marked.

Parallelizing using way A, by fixing the cut-off limits of crDR to <1350 deaths per 100,000 for noMMC and >800 deaths per 100,000 for MMC, the difference in crDR between both groups became 0.5 deaths per 100,000 (926.2 vs 925.7) which resulted in adequate parallelization of the groups.

**Figure 4:**
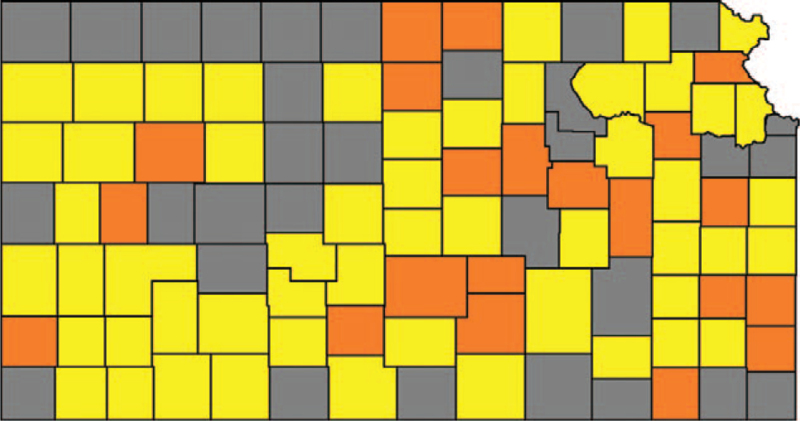
Kansas counties included in the analysis, configuration A. Mask-mandated counties (MMC) are orange, counties without mask mandate (noMMC) are yellow. Grey counties were excluded.

These cut-off limits eliminated 31 counties (mostly small counties from the noMMC category) and 41.3% of the population (mostly from the MMC category). Note that Sedgwick County with 516,042 people and an crDR of 802.5 deaths per 100,000 got narrowly included in the analysis. Figure 4 shows the counties after step 2A.

Parallelizing using way B, by fixing the cut-off limits of crDR to >805 for MMC and >600 for noMMC, the difference in crDR between both groups became 8.7 deaths per 100,000 (less than one percent) which also resulted in adequate parallelization of the groups.

**Figure 5:**
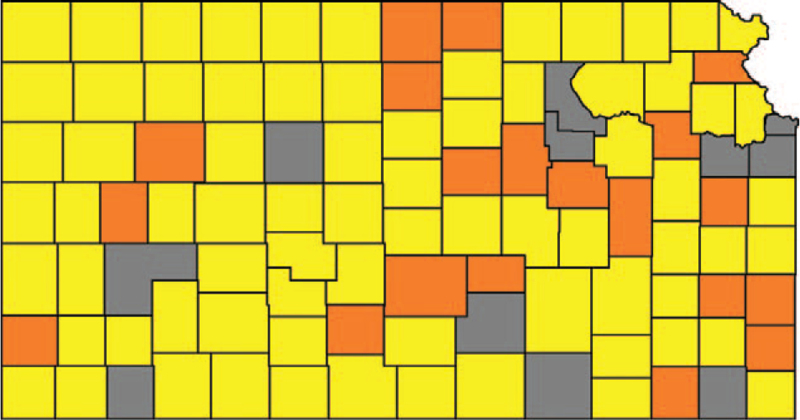
Kansas counties included in the analysis, configuration B. Mask-mandated counties (MMC) are orange, counties without mask mandate (noMMC) are yellow. Grey counties were excluded.

These cut-off limits eliminated only 11 counties but 56.7% of the population. Figure 5 shows the counties after step 2B.

The names of these final counties and their corresponding group are shown for both configurations in the Supplemental Digital Content Appendix.

### 3.3 Step 3: Analyzing the data

The results for both configurations are shown in Table 2.

**Table 2:** Results of the analysis (step 3)^∗^.

	Configuration A		Configuration B	
	MMC^†^	noMMC^‡^	MMC^†^	noMMC^‡^
People total	1,072,139	638,955	556,097	704,210
Infected people	13,655	9880	7,563	10,403
Deaths	241	95	156	137
CFR^§^	1.76%	0.96%	2.06%	1.32%
RR^¶^ (MMC^†^)	1.85 [1.51–2.10]		1.58 [1.34–1.84]	
p	<0.001		<0.001	
CFR_G_^§^	1.69%	0.96%	1.93%	1.32%
RR_G_^¶^ (MMC^†^)	1.77 [1.45–2.01]		1.48 [1.25–1.73]	
p_G_	<0.001		0.001	

† mask mandated counties

‡ counties without mask mandate

§ case fatality rate

¶ risk ratio.

∗ Subscript G indicates correction for the outlier of Gove County.

To correct for the CFR outlier of Gove County, the number of deaths in Gove County was reduced from 13 to 3, as marked by the subscript “G.”

Furthermore, a sensitivity analysis was performed by excluding counties without a mask mandate that had counties with a mask mandate, as shown in Table 3, which did confirm the prior results.

**Table 3:** Sensitivity analysis (step 3), excluding counties without mask mandate with cities with mask mandate.

	Configuration A		Configuration B	
	MMC^†^	noMMC^‡^	MMC^†^	noMMC^‡^
People total	1,038,944	604,718	522,902	658,089
Infected people	13,138	9,503	7036	9970
Deaths	214	93	130	135
CFR^§^	1.63%	0.98%	1.85%	1.35%
RR^¶^ (MMC^†^)	1.68 [1.37–1.91]		1.37 [1.16–1.63]	
p	<0.001		0.01	

† mask mandated counties

‡ counties without mask mandate

§ case fatality rate

¶ risk ratio

### 3.4 Step 4a: Infection rate correlated bias check (optional)

As the RR was significant and infection rate in noMMC was higher, an additional test was performed to examine protection bias.

The results are shown in Table 4.

**Table 4:** Results after infection rate correlated bias check (Step 4a)^∗^.

	Configuration A		Configuration B	
	MMC^†^	noMMC^‡^	MMC^†^	noMMC^‡^
People total	1,072,139	638,955	556,097	704,210
Infected (corr.)	16,578	9,880	8215	10,403
Deaths	241	95	156	137
CFR^§^	1.45%	0.96%	1.90%	1.32%
RR^¶^ (MMC^†^)	1.52 [1.24–1.72]		1.45 [1.23–1.69]	
p	<0.001		0.001	
CFR_G_^§^	1.39%	0.96%	1.78%	1.32%
RR_G_^¶^ (MMC^†^)	1.46 [1.19–1.65]		1.36 [1.15–1.59]	
p_G_	0.002		0.01	

† mask mandated counties

‡ counties without mask mandate

§ case fatality rate

¶ risk ratio.

∗ Subscript G indicates correction for the outlier of Gove County.

### 3.5 Step 4b: Confounder check (optional)

The additional deaths among those infected in MMC was 111 (95% CI 82–126) in configuration A respectively 57 (95%CI 39–71) in configuration B. If these deaths (among infected individuals) were not related to COVID-19, 17,031 (95% CI 12,582–19,333) and 15,802 (95% CI 10,812–19,683) additional deaths among non-infected individuals would be expected in configurations A and B, respectively.

According to CDC, the average number of expected all-cause deaths in Kansas from August 2nd to November 7th 2020 was 6867 (98 days compared to the study's 76 days). The number of deaths without COVID-19 during this time span was 7382, resulting in 515 excess deaths not related to COVID-19.

Comparing these 515 excess deaths to the numbers of expected additional deaths (where even the lower CI are over 10,300), this means that non-COVID factors (i.e., possible confounders) represent less than 5.0% (515/10,300) of the RR increase, thus looking at other factors that would reduce that percentage even further (noMMC counties among excess deaths and adjusting for the different timespan mentioned above) was unnecessary.

### 3.6 Step 4c: Negative control (optional)

There was no statistically significant difference between case fatality rates from February 1st, 2020 to April 15th, 2020 in neither configuration (Configuration A: *P* = .86; RR = 1.06 [0.65–1.56], configuration B: *P* = .64; RR = 1.2 [0.73–2.02]).

Furthermore, Table 5 demonstrates the change of RR under the assumption that of 15% of deaths were not caused by severe complications of COVID-19 as underlying cause of death.^[15]^

**Table 5:** Adjusting RR for a rate of 85% of deaths with COVID-19 as underlying cause.

No-MMC^†^	MMC^‡^	
9880	13665	Infected
95	241	Deaths
0	111	Thereof additional deaths by mask mandate^§^
73	101	85% deaths with COVID-19^¶^ as underlying cause of death without mask mandate^††^
73	212	deaths with COVID-19 as underlying cause of death^‡‡^
0,74%	1,55%	CFR^§§^ (for deaths with COVID-19 as underlying cause of death)
	2,10	RR^¶¶^ (for deaths with COVID-19 as underlying cause of death)

† counties without mask mandate

‡ mask mandated counties.

§ As calculated in step 4b

¶ coronavirus disease 2019.

†† ([total deaths] ^∗^ 85% − [additional deaths by mask mandate]) ^∗^ [infected in group]/ [infected total].

‡‡ Total of the two rows above

§§ case fatality rate

¶¶ risk ratio (MMC/noMMC).

## 4 Discussion

The objective of this study was to find out whether mask mandates contribute to the COVID-19 CFR by comparing data between Kansas counties that had mask mandates and those that did not have mask mandates during the same time period in the summer of 2020.

The most important finding from this study is that contrary to the accepted thought that fewer people are dying because infection rates are reduced by masks, this was not the case. Results from this study strongly suggest that mask mandates actually caused about 1.5 times the number of deaths or ∼50% more deaths compared to no mask mandates. This means that the risk for the individual wearing the mask should even be higher, because there is an unknown number of people in MMC who either do not obey mask mandates, are exempted for medical reasons or do not go to public places where mask mandates are in effect. These people do not have an increased risk and thus the risk on the other people under a mask mandate is actually higher.

The mask mandates themselves have increased the CFR by 1.85 / 1.58 or by 85% / 58% in counties with mask mandates. It was also found that almost all of these additional deaths were attributed solely to COVID-19. Therefore, this number is most likely underestimated and depends to a large extent on the percentage of people who tested positive for SARS-CoV-2 but did not die with COVID-19 as the underlying cause of death. The study by Cobos-Siles et al^[15]^ described that 15% of patients with COVID-19 infection died from decompensation due to other pathologies and the cause of death was unrelated to severe complications of COVID-19. The study by Rommel et al^[16]^ describes that from 38.641 deaths with and by COVID-19 only 31.638 (81.9%) were reported with COVID-19 as the underlying cause of death. Correcting for this phenomenon (using the former value by Cobos-Siles) raises the RR for deaths with COVID-19 as the underlying cause to 2.10 (in configuration A).

### 4.1 Hypothesis

A rationale for the increased RR by mandating masks is probably that virions that enter or those coughed out in droplets are retained in the facemask tissue, and after quick evaporation of the droplets,^[17]^ hypercondensed droplets or pure virions (virions not inside a droplet) are re-inhaled from a very short distance during inspiration. This process will be referred to as the “Foegen effect” because a review of the literature did not yield any results on this effect, which has not been described earlier.

The fundamentals of this effect are easily demonstrated when wearing a facemask and glasses at the same time by pulling the upper edge of the mask over the lower edge of the glasses. Droplets appear on the mask when breathing out and disappear when breathing in.

In the “Foegen effect,” the virions spread (because of their smaller size) deeper into the respiratory tract.^[18]^ They bypass the bronchi and are inhaled deep into the alveoli, where they can cause pneumonia instead of bronchitis, which would be typical of a virus infection. Furthermore, these virions bypass the multilayer squamous epithelial wall that they cannot pass into in vitro^[19]^ and most likely cannot pass into in vivo. Therefore, the only probable way for the virions to enter the blood vessels is through the alveoli.

Moreover, the “Foegen effect” could increase the overall viral load because virions that should have been removed from the respiratory tract are returned. Viral reproduction in vivo, including the reproduction of the re-inhaled virions, is exponential compared with the mask-induced linear droplet reduction.^[20]^ Therefore, the number of exhaled or coughed out virions that pass through the facemask might, at some point, exceed the number of virions shed without facemasks. Furthermore, the hypercondensed droplets and pure virions in the mask might be blown outwards during expiration, resulting in aerosol transmission instead of droplet transmission. Moreover, these 2 effects might be linked to a resurgence of rhinovirus infections.^[21]^

The use of “better” masks (e.g., FFP2, FFP3) with a higher droplet-filtering capacity probably should cause an even stronger “Foegen effect” because the number of virions that are potentially re-inhaled increases in the same way that outward shedding is reduced.

Another salient point is that COVID-19-related long-term effects and multisystem inflammatory syndrome in children may all be a direct cause of the “Foegen effect.” Virus entry into the alveoli and blood without being restricted to the upper respiratory tract and bronchi and can cause damage by initiating an (auto) immune reaction in most organs.

Regarding the proposed consequences of the “Foegen effect,” the question arises which share of the global death toll and long-term effects of COVID-19 can be attributed to widespread mask use.

### 4.2 Supporting literature

Chan et al^[22]^ proved the “Foegen effect” in a golden Syrian hamster by showing a significant increase in viral load in the lungs of masked hamsters compared with non-masked hamsters (*P* < .05). Unfortunately, these findings are left undiscussed in their study. As their study also finds an increased viral load in the lung when only the infected hamster was masked, this reinforces the abovementioned theory of the facemasks increasing the number of aerosols emitted by the wearer.

The study by Adjodah et al^[23]^ analyzes the effect of mask mandates on cases and mortality (but not CFR) in the USA on a pre-post-basis, and finds that after the lifting of a mask mandate, cases rise but mortality does not, which effectively means that lifting a mask mandate lowers the CFR. Conversely, the implementation of a mask mandate increases CFR. This can also be seen in the data from Adjodah et al by taking the delay between infection and death (14 days^[11]^) into account: Deaths on day 40 are still within the 95% CI of day 14, while cases on day 26 are significantly lower (compared to day zero).

While 1 might think that obstructing the expiratory pathway in respiratory infections has never been performed before, this is regularly performed for patients with acute respiratory distress syndrome, wherein ventilation masks, and not facemasks, are provided to increase the oxygen supply. Frat et al^[24]^ compared ventilation masks to nasal cannulas and showed a significant difference that favoured nasal cannula use based on a 90-day mortality assessment. Patel et al^[25]^ compared ventilation masks to an airtight but ventilated helmet around the patient's head; however, the trial was stopped early based on the predefined criteria for efficacy: the mask group had significantly less ventilator-free days, a worse intubation rate, and higher overall mortality, which was attributed to a slightly higher positive end-expiratory pressure in the ventilation mask group; however, a meta-analysis by Guo et al^[26]^ showed that a high positive end-expiratory pressure correlated with a better outcome, making the helmet study an important indication of the existence of the “Foegen effect.”

Improved respiratory clearance using mucoactive agents, such as herbal medicines^[27]^ that have been used for centuries or newly developed pharmaceutical drugs,^[^28^,^^29^^]^ compared with a placebo, reduces exacerbations of respiratory tract infections. Certain observations during the ongoing COVID-19 pandemic, especially the high death rate among medical personnel in Italy during the “first wave” of the pandemic,^[30]^ could be attributed to working for many hours while wearing facemasks, despite being ill. The accumulation of virions in facemasks was demonstrated by Chughtai et al.^[31]^

### 4.3 Limitations and scope

The main confounder of old age and illness has been accounted for by the parallelisation approach. Comparing counties within one state also leads to minimal differences in access to and quality of the health system, testing numbers, culture and behaviour regarding health and mask usage, climate, and time of infection peaks. This and the use of 2 different configurations (see Tables 2, 3 and 4) means that there is no systematic confounding in these overall much weaker confounding factors.

This study was based on secondary data analysis; thus, future studies with a prospective design are required to understand this research question more clearly.

Ethical principles prevent clinical studies to be conducted to prove the “Foegen effect” in vivo in humans and wearing a mask is not blindable; thus, proving the “Foegen effect” further in humans may be very difficult, especially considering the results of the helmet trial^[25]^ and early termination as the results for the mask group were extremely poor.

However, a sick person breathing out through a mask (without inhaling) and a puppet “inhaling” through that same mask into a particle collector shortly thereafter might help prove the “Foegen effect.”

Another method of proving or disproving the “Foegen effect” is the use of (H_2_O)-O-15 positron emission tomography. The proband will gargle with (H_2_O)-O-15, spit it out, then either put on a mask or not, take some deep breaths in and out, and then measurements of thorax and head are started immediately. As by the “Foegen effect,” the positron emission tomography scan should show (more) water being inhaled into the lungs.

Furthermore, lung radiographs were particularly shadowed in the lower lobe and peripherally at the beginning of the pandemic,^[32]^ but there are unconfirmed observations by healthcare professionals that now, in the wake of the mask requirement, the shadowing is ubiquitous. A corresponding retrospective study could relate the degree of shadowing (and thus the severity of the infection) to the time of average mask wearing.

In animal models, the “Foegen effect” was observed in a golden hamster model. Research on other animals, especially rhesus monkeys, should be conducted. However, it is important to note that the effect was observed on day 5 post-challenge, but not on day 7. This indicates that the duration of the effect is shorter in healthy individuals, which is plausible because the overall access of immune cells to alveolar epithelium is better than that to the epithelium of the oropharynx. Thus, when testing the “Foegen effect” in animals, multiple endpoints for sacrifice (e.g., daily) should be considered.

Further research should quantify the number of non-COVID-19-related deaths, both in populations with and without mask mandates, to understand the full extent of the effect on CFR. The consequences of the “Foegen effect” in aerosol transmission and viral load on infection rates should be evaluated in future research.

## 5 Conclusion

This study revealed that wearing facemasks might impose a great risk on individuals, which would not be mitigated by a reduction in the infection rate. The use of facemasks, therefore, might be unfit, if not contraindicated, as an epidemiologic intervention against COVID-19. Proving or disproving the “Foegen effect” using experimental studies as described above should be a priority to public health scientists.

## Acknowledgments

I am grateful for the helpful comments by Prof. Oliver Hirsch. I would like to thank Editage [http://www.editage.com] for the scientific editing of this manuscript as well as for editing and reviewing it for English language.

## Author contributions

**Conceptualization:** Zacharias Fögen.

**Data curation:** Zacharias Fögen.

**Formal analysis:** Zacharias Fögen.

**Funding acquisition:** Zacharias Fögen.

**Investigation:** Zacharias Fögen.

**Methodology:** Zacharias Fögen.

**Project administration:** Zacharias Fögen.

**Resources:** Zacharias Fögen.

**Software:** Zacharias Fögen.

**Supervision:** Zacharias Fögen.

**Validation:** Zacharias Fögen.

**Visualization:** Zacharias Fögen.

**Writing – original draft:** Zacharias Fögen.

**Writing – review & editing:** Zacharias Fögen.

## Supplementary Material

Supplemental Digital Content
