# Supplementary material for: The Foegen effect: A mechanism by which facemasks contribute to the COVID-19 case fatality rate
Source: Medicine (Baltimore). 2022 Feb 18;101(7):e28924. doi: 10.1097/MD.0000000000028924 (PMC9282120; doi:10.1097/MD.0000000000028924)
Supplement: Supplemental Digital Content [file medi-101-e28924-s001.docx]

**Supplementary material – Groups after step 2**

**Configuration A**

**mask mandated counties (MMC):**

Allen, Atchison, Bourbon, Crawford, Dickinson, Franklin, Gove, Harvey, Jewell, Lyon, Mitchell, Montgomery, Morris, Pratt, Reno, Republic, Saline, Scott, Sedgwick, Shawnee, Stanton

**counties without mask mandate (noMMC):**

Anderson, Barber, Barton, Butler, Chase, Clark, Clay, Coffey, Doniphan, Edwards, Elk, Ellsworth, Finney, Ford, Graham, Grant, Gray, Hamilton, Haskell, Jackson, Jefferson, Kearny, Kingman, Kiowa, Leavenworth, Lincoln, Linn, Logan, McPherson, Meade, Miami, Nemaha, Neosho, Osage, Osborne, Ottawa, Pawnee, Pottawatomie, Rice, Seward, Sheridan, Sherman, Stafford, Stevens, Sumner, Thomas, Trego, Wabaunsee, Wallace, Washington, Wichita, Wilson, Woodson

**Configuration B**

**mask mandated counties (MMC):**

Allen, Atchison, Bourbon, Crawford, Dickinson, Franklin, Gove, Harvey, Jewell, Lyon, Mitchell, Montgomery, Morris, Pratt, Reno, Republic, Saline, Scott, Shawnee, Stanton

**counties without mask mandate (noMMC):**

Anderson , Barber , Barton , Brown , Butler , Chase , Chautauqua , Cherokee , Cheyenne , Clark , Clay , Clay , Coffey , Comanche , Decatur , Doniphan , Edwards , Elk , Ellsworth , Ford , Graham , Grant , Gray , Greeley , Greenwood , Hamilton , Harper , Haskell , Hodgeman , Jackson , Jefferson , Kearny , Kingman , Kiowa , Lane , Leavenworth , Lincoln , Linn , Logan , Marion , Marshall , McPherson , Meade , Miami , Morton , Nemaha , Neosho , Ness , Norton , Osage , Osborne , Ottawa , Pawnee , Phillips , Pottawatomie , Rawlins , Rice , Rocks , Rush , Russell , Sheridan , Sherman , Smith , Stafford , Stevens , Sumner , Thomas , Trego , Wabaunsee , Wallace , Washington , Wichita , Wilson , Woodson
